# Multifocal Retroperitoneal Sarcoma

**DOI:** 10.1155/2013/763702

**Published:** 2013-05-02

**Authors:** Theodosios Theodosopoulos, Dionysios Dellaportas, Vasiliki Psychogiou, Anneza Yiallourou, George Polymeneas, Georgios Gkiokas, Dionysios Voros

**Affiliations:** 2nd Department of Surgery, University Hospital “Aretaieion”, 115 28 Athens, Greece

## Abstract

*Introduction*. Retroperitoneal sarcomas comprise a small proportion of all soft tissue sarcomas, and multiple factors influence their clinical behavior. Histopathological type and grade as well as complete surgical resection especially on the first operative attempt are well recognized as the main prognostic factors. Multifocality is another prognostic factor, which compromises therapy and finally makes prognosis worse due to multiple adverse implications. *Case Presentation*. A rare case of a 65-year-old male patient suffering from a multifocal retroperitoneal liposarcoma successfully treated in our hospital is presented herein. *Discussion*. Also, general considerations for these tumors are discussed, and especially multifocality is underlined as an ominous sign of retroperitoneal sarcomas behavior. Despite multifocality, once again complete surgical excision remains the mainstay of treatment of these patients, as long as further systemic and local therapies do not provide durable results.

## 1. Introduction

Sarcomas are uncommon neoplasms of mesenchymal origin accounting for 1% of all solid tumors, while retroperitoneal sarcomas (RPS) are even less often found accounting for 10% of all soft tissue sarcomas [1]. They comprise a heterogeneous group of various histopathologic subtypes, and liposarcomas, leiomyosarcomas, and malignant fibrous histiocytomas are the commonest found in the retroperitoneal space [2]. Multiple factors such as complete surgical resection and histopathologic type and grade influence prognosis of these challenging in the management of tumors. Another ominous sign often not highlighted enough is multifocality [3, 4]. The latter is defined as having more than one tumor and has been associated with poor prognosis and higher recurrence rates. This feature obviously complicates management options of these patients. We report a rare case of a 65-year-old male patient with four different foci of retroperitoneal liposarcoma at the time of diagnosis and its management. 

## 2. Case Presentation

**Figure 1:**
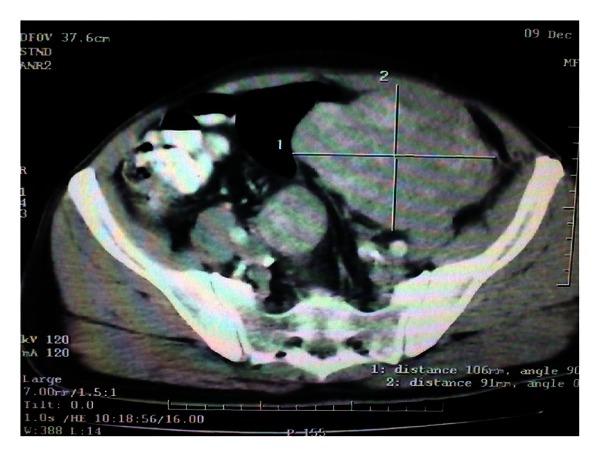
CT scan depicting the left and right iliac fossa mass of the patient.

**Figure 2:**
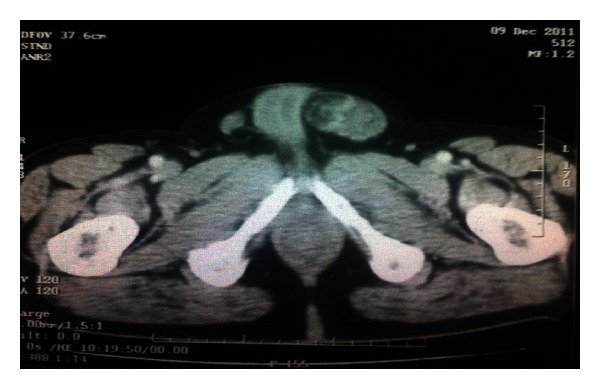
CT scan showing the left inguinal mass.

**Figure 3:**
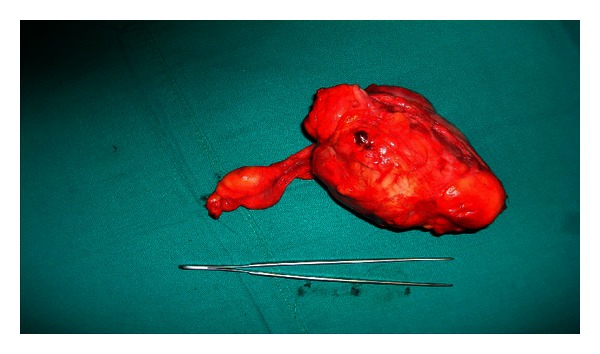
Surgical specimen which includes the right and left iliac fossa masses.

A 65-year-old white male complaining of mild abdominal discomfort and nonspecific abdominal pain, as well as a swelling in his left inguinal area, underwent an abdominal computed tomography (CT) scan (Figure 1). A large mass in his left iliac fossa measuring 11 × 10.5 × 9 cm and another one in his right iliac fossa measuring 4.3 cm were revealed, as well as another solid and individual mass measuring 6 × 5.5 × 2.2 cm in his left inguinal area (Figure 2). Afterwards and because of the possible sarcomatous lesion being the most probable scenario, he underwent a thorax CT scan which showed no signs of metastatic disease. The patient also had 6 months prior to his admission an angioplasty and stent placement for coronary disease, and he was under anticoagulant agents. He was first managed in a district hospital where the left inguinal mass causing the greatest discomfort was excised and turned to be a well-differentiated liposarcoma. He was referred to our hospital, and operative intervention was decided as the only durable treatment option. The patient had a midline laparotomy with concomitant radical excision of the other two masses of the left and right iliac fossa, which were histopathologically, and liposarcomas as well. It has to be mentioned that the left iliac fossa mass had a separate satellite 4 cm stalk (Figure 3). He had an uneventful postoperative course, and he was discharged on the 6th postoperative day. No adjuvant treatment was decided, and he remains-disease free 14 months later.

## 3. Discussion

The overall incidence of RPS is 0.3-0.4% per 100,000 of the population, and usually patients are on their fifth to sixth decade of life [5]. Retroperitoneal sarcomas are silent tumors often growing large before being detected by the patient or the clinician and many times are revealed in imaging for unrelated reasons [6]. Their most typical manifestations are discomfort or nonspecific abdominal pain and a palpable abdominal mass, while one-third of patients will have some distal neurologic sign or symptom from the mass effect. Sometimes a low-grade fever may develop due to central tumor necrosis, and rarely erosion of adjacent organs may lead to gastrointestinal bleeding [7]. 

Approximately, 11% of patients who have a primary retroperitoneal sarcoma also present with metastatic disease, with lung mostly and liver secondly, being the most common site of metastasis. Imaging is of paramount importance because staging, surgical plan, and sometimes image-guided biopsy are based on it. Abdominal CT scan is the most important tool for demonstrating an RPS and its relation with vital structures, great vessels, and neighboring organs that may need to be resected en bloc, and for the case of liposarcoma, can predict the histologic type and even the grade of the tumor [8]. CT scan is the main modality for staging, and MRI is reserved for better defining intermediate liver lesions and administering questions of vascular invasion. 

Treatment is based on surgical resection as other options like chemotherapy and radiotherapy have poor results and can be regarded only in adjuvant and rarely neoadjuvant setting. In various series, complete resection rates vary from 54–93% [9]. Avoidance of tumor spillage intraoperatively is very important for reducing local recurrence rates, and en bloc adjacent organ resection is based on that effort and varies by series from approximately 34–75%. Tumor involvement of the adjacent organ is seldom if ever demonstrated, and the most common resected organs are the kidney, small and large bowel, the ureter, the bladder, and pelvic organs of the female genital tract. Retroperitoneal approach through a paramedian incision is avoided in large tumors as in our case in order to have better mobilization of adjacent structures and en bloc resection of involved structures. The therapeutic goal is the complete resection with healthy macroscopic and microscopic margins (R0 surgical excision). 

However, even when RPS is surgically completely resectable (R0), the main challenge is the high rate of local recurrence, which results in poor overall survival [10]. In recent large series of complete resection, the five-year disease-free survival rate was only 18% [4]. One obvious reason for local recurrence except for incomplete resection microscopically or macroscopically (R1 or R2), which is excluded by thorough histopathological study of the surgical specimen, is multifocality of these tumors not detected by preoperative imaging modalities as much as not found intraoperatively. 

In a previous report from our institution, the incidence of multifocal disease was 20.3% for primary RPS which is comparable with a recent series from MD Anderson Cancer Center [4, 11]. Multifocality in RPS is considered an independent factor for poor overall survival. The main reason for that is that multifocal disease can be spread throughout the retroperitoneal fat and can be missed either during preoperative imaging usually with CT scan or intraoperatively. The remaining foci could be the main reason for local recurrence and poor outcome for these patients. There are also previous reports underlining multifocal disease as an adverse and not so uncommon characteristic of RPS in contrast with extremity sarcomas [12, 13]. Despite being an ominous prognostic factor, multifocal RPS should be considered for surgical intervention, as in those patients where R0 resection is achieved, long term survival is observed. 

For all the above, resection of RPS through an extended laparotomy with wide en bloc excision of surrounding soft tissues seems absolutely necessary. The existence of “satellite tumors” on the preoperative CT scan may be used as a guide for appropriate surgical approach and an extension of resection and may serve as a prognostic indicator [3].
